# Gratitude and savoring interventions for older adults: a narrative review

**DOI:** 10.1093/geront/gnag206

**Published:** 2026-08-27

**Authors:** Noortje Kloos, Rebecca Baxter, David Edvardsson, Debby Gerritsen, Ernst Bohlmeijer

**Affiliations:** Department of Primary and Community Care, Radboud Institute for Health Sciences, Radboudumc Alzheimer Center, University Knowledge Network for Older Adult Care Nijmegen (UKON), Radboud University Medical Center, Nijmegen, The Netherlands; Centre for eHealth and Well-Being Research, Department of Psychology, Health and Technology, University of Twente, Enschede, The Netherlands; Department of Nursing, Umeå University, Umeå, Sweden; Department of Nursing, Swinburne University, Melbourne, Victoria, Australia; Gothenburg Centre for Person-Centred Care, Institute of Health and Care Sciences, Sahlgrenska Academy, University of Gothenburg, Gothenburg, Sweden; Department of Primary and Community Care, Radboud Institute for Health Sciences, Radboudumc Alzheimer Center, University Knowledge Network for Older Adult Care Nijmegen (UKON), Radboud University Medical Center, Nijmegen, The Netherlands; Centre for eHealth and Well-Being Research, Department of Psychology, Health and Technology, University of Twente, Enschede, The Netherlands

**Keywords:** Positive psychology, Well-being, Dementia, Mental health

## Abstract

**Background and Objectives:**

Gratitude and savoring have great potential to support well-being in older age, but most studies on positive psychology interventions have been conducted among healthy young adults. This narrative review explored gratitude and savoring interventions for older adults, with or without dementia, including (a) the characteristics of these interventions, (b) their acceptability for older users, and (c) their effect on mental health.

**Research Design and Methods:**

Scopus, PubMed, PsychInfo, Web of Science, and CINAHL were searched in December 2023 and April 2025. Studies were eligible if they examined gratitude or savoring interventions among older adults. Multi-component intervention studies were excluded. 2 researchers independently screened 2,423 studies and assessed methodological quality using the Mixed Methods Appraisal Tool. Numerical and narrative data were extracted and synthesized regarding intervention characteristics, acceptability, and effectiveness.

**Results:**

Of the 11 included articles, 5 examined gratitude, 5 examined savoring, and 1 compared both. Only 1 study included participants with dementia. Adherence was generally high, suggesting a good fit, but participant evaluation was lacking. 4 acceptability themes were highlighted concerning the target group, setting and delivery mode, dosage, and content and tailoring. Most studies showed improved well-being and reduced psychopathology, although not consistently on all outcome measures.

**Discussion and Implications:**

There is initial evidence that gratitude and savoring interventions could be suitable to promote mental health among older adults with relatively preserved cognitive function. Future research should examine the need for tailoring in terms of dose, delivery method and content, and applicability to more diverse groups of older adults.

## Background and objectives

Positive psychology interventions (PPIs) have been developed to support people to focus on the positive aspects of life and have been shown to enhance mental health by improving well-being and reducing psychopathology (Bolier et al., 2013; Carr et al., 2024; Chakhssi et al., 2018; Hendriks et al., 2018, 2020; Sin & Lyubomirsky, 2009; Weiss et al., 2016). However, most of the studies on the effectiveness of PPIs thus far have considered healthy younger adults (Carr et al., 2021), with little focus on their impact among older adults or persons with dementia. The aim of the current review is to shed light on the present state of gratitude and savoring interventions as two quintessential positive psychology pathways to improving well-being (Schueller et al., 2014) that have potential to support older adults with and without dementia.

Attending to the positive things in life elicits positive emotions, such as love, joy and serenity, which seem to undo the negative effects of negative emotions to some extent (Behnke et al., 2023; Fredrickson, 2001), and can act as a buffer against stress symptoms, such as anxiety, and anger (e.g., Demorest, 2020; Zander-Schellenberg et al., 2020). The broaden-and-build theory describes how experiencing positive emotions broadens attentional awareness and increases flexibility and exploratory behaviors, which builds enduring personal resources (Fredrickson, 2013). This, in turn, improves mental health and increases the odds of experiencing subsequent positive emotions, creating an upward spiral (Fredrickson, 2013).

Two ways of attending to the positive things in life are gratitude and savoring exercises (Schueller et al., 2014). Gratitude refers to appreciating the good aspects of life and the goodness of others (Sansone & Sansone, 2010). Gratitude can be viewed as a positive moral emotion or mood in reaction to receiving perceived benefits from someone (McCullough et al., 2002, 2004), or a general dispositional tendency (Watkins et al., 2003; Wood et al., 2010). Interventions aimed at cultivating gratitude, such as gratitude journaling, or writing a gratitude letter (Emmons & McCullough, 2003; Emmons & Stern, 2013), have shown to improve mental health in various adult samples (e.g., Bohlmeijer et al., 2021; Kloos et al., 2022).

Savoring is the cognitive process of attending to positive experiences and intentionally engaging in thoughts and behaviors to promote the generation, maintenance, and enhancement of positive affect (Bryant & Veroff, 2007; Bryant et al., 2011). During the savoring process, one is aware of pleasant events and the positive feelings that are experienced. In this way, savoring can increase the intensity, duration, and frequency of positive feelings (Bryant & Veroff, 2007). Savoring can be focused on recalling positive past events, on present experiences, or on anticipating imminent positive events (Bryant & Veroff, 2007), and is related to positive mental health outcomes (e.g., Biskas et al., 2019; Pereira et al., 2021).

Although gratitude and savoring are conceptually distinct constructs, they share a central feature that provides the rationale for considering them together. Both involve directing attention toward positive aspects of experience and fostering appreciation of these experiences. Savoring refers broadly to the process of attending to, appreciating, and potentially enhancing positive experiences, whether they are anticipated, occurring in the present, or recalled from the past. Gratitude, by contrast, specifically involves recognizing and appreciating valued benefits received from another person or external source, giving it a stronger interpersonal and moral dimension (McCullough et al., 2001; Watkins 2014; Wood et al., 2008). Despite these differences, many interventions designed to promote gratitude or savoring employ similar strategies that encourage individuals to notice and appreciate positive experiences in daily life. Consequently, the boundary between gratitude and savoring interventions is not always clear-cut, which is also reflected in divergent findings regarding their relationship in prior research suggesting savoring may encompass or regulate gratitude (Bryant et al., 2011; Lauzon & Green-Demers, 2020), moderate it (Hu et al., 2024), or represent a distinct construct with a different temporal focus (Burzynska-Tatjewska et al., 2022a, 2022b). In the present review, we therefore considered both intervention types while recognizing their conceptual distinctions and categorized interventions according to their primary intended psychological process.

Gratitude and savoring may be especially relevant for older adults. Socio-emotional selectivity theory describes how the awareness of limited time leads older adults to prioritize established relationships and positivity (Carstensen et al., 1999*)*, which offers greater opportunity for reciprocal altruism and expressions of gratitude. Besides, because of their awareness of mortality, older people may also be particularly able to savor the past and present moments, and appreciate the good things (Kurtz, 2008; Layous et al., 2018). Importantly, there is some indication that PPIs may have greater effects on the mental health of older than of younger participants (Carr et al., 2021). For instance, a meta-analysis of gratitude interventions showed larger effects for adult participants compared to children and college-age participants (Dickens, 2017). However, specific effects for older adults could not be isolated. One systematic review that examined various PPIs specifically for healthy older adults found promising effects on mental health (Sutipan et al., 2017). This review included mostly reminiscence interventions and multi-component interventions, but the authors specifically noted the potential of a gratitude intervention (recording three good things daily) on durable well-being promotion of older adults (Killen & Macaskill, 2015).

At the same time, the evidence base for gratitude and savoring interventions specifically for older adults remains limited and inconclusive. A systematic review on randomized controlled trials of psychological interventions targeting well-being in healthy older adults found no studies explicitly focusing on gratitude or savoring interventions (Iwano et al., 2022). Reviews focusing more broadly on adult populations, including older adults, suggested mixed weak effects for savoring-related interventions (only life review and reminiscence) in older adult clinical samples (Cullen et al., 2024), whereas gratitude interventions appeared to have beneficial effects, although age-specific effects could not be disentangled (Diniz et al., 2023). Importantly, both reviews included only randomized controlled trials.

Given the growing number of PPI studies (Carr et al., 2024), it is timely to explore the relevance and effectiveness of gratitude and savoring interventions for older adults across a range of study designs. The applicability of these interventions for older adults living with dementia is of particular interest, given that they represent a rapidly growing segment of the aging population (WHO, 2017). This narrative review aimed to cover gratitude and savoring interventions for older adults, with or without dementia. Three review questions were formulated:

1. What are the characteristics of savoring and gratitude interventions for older adults?
2. How acceptable are gratitude and savoring interventions for older users?
3. How effective are these interventions for improving the mental health of older adults?

## Research design and methods

### Design

The study used a narrative review design with a systematic search (Sutton et al., 2019) to investigate intervention characteristics, acceptability, and effectiveness of savoring and gratitude interventions for older adults. This review was registered in PROSPERO (registration record CRD42023487496) and reported in accordance with the PRISMA guidelines (Page et al., 2021).

### Eligibility criteria

Inclusion criteria were defined using PICO framework (Population, Intervention, Comparison, Outcomes; Eriksen & Frandsen, 2018): *Population* included older adults (i.e., either authors of primary studies describing their population as “older adults,” or the sample included mainly people aged ≥60) with and without dementia living both in the community and in (long-term) care facilities (e.g., nursing home; hospital; hospice), *Intervention* concerned gratitude and savoring interventions, intervention studies with and without *Comparison groups* were included, and *Outcomes* comprised intervention characteristics, acceptability, and effectiveness of mental health outcomes (both *well-being*: positive emotional, social and psychological functioning, and *psychopathology*: stress, depression, and anxiety symptoms; Bohlmeijer & Westerhof, 2021). Only peer-reviewed studies published in English were included.

Biological and pharmacological interventions were excluded. We also decided to exclude multi-component PPIs with a gratitude or savoring exercise, as the studies commonly do not report acceptability and effectiveness of these specific elements.

### Search strategy

We conducted a systematic literature search in the electronic databases Scopus, PubMed, PsycINFO, Web of Science, and CINAHL. The first author (N.K.) and second author (R.B.) developed the search string for each database with support from a librarian, combining the terms and synonyms of [“gratitude” or “savoring” or “positive psychology”] AND [intervention] AND [“older adult” or “dementia”] (see Table S1). Databases were searched on title, and on abstract and keywords where applicable. A research associate (M.W.) conducted the initial search on December 4, 2023. A final search was conducted on April 4, 2025 to update the literature. The reference lists of the included studies and previous reviews on gratitude and savoring were also searched, but this did not lead to additional inclusions.

### Screening procedure

The review management platform Covidence systematic review software (2024) was used to screen the studies. References from the five databases were combined and duplicates were removed automatically. The first and second authors (N.K., R.B.) independently screened the article titles and abstracts against the eligibility criteria. The full texts of potentially eligible studies were independently screened by the first author (N.K.) and a research associate (M.W.). Disagreements were discussed until consensus was reached. Figure 1 shows the PRISMA flow diagram of the study selection process.

**Figure 1:**
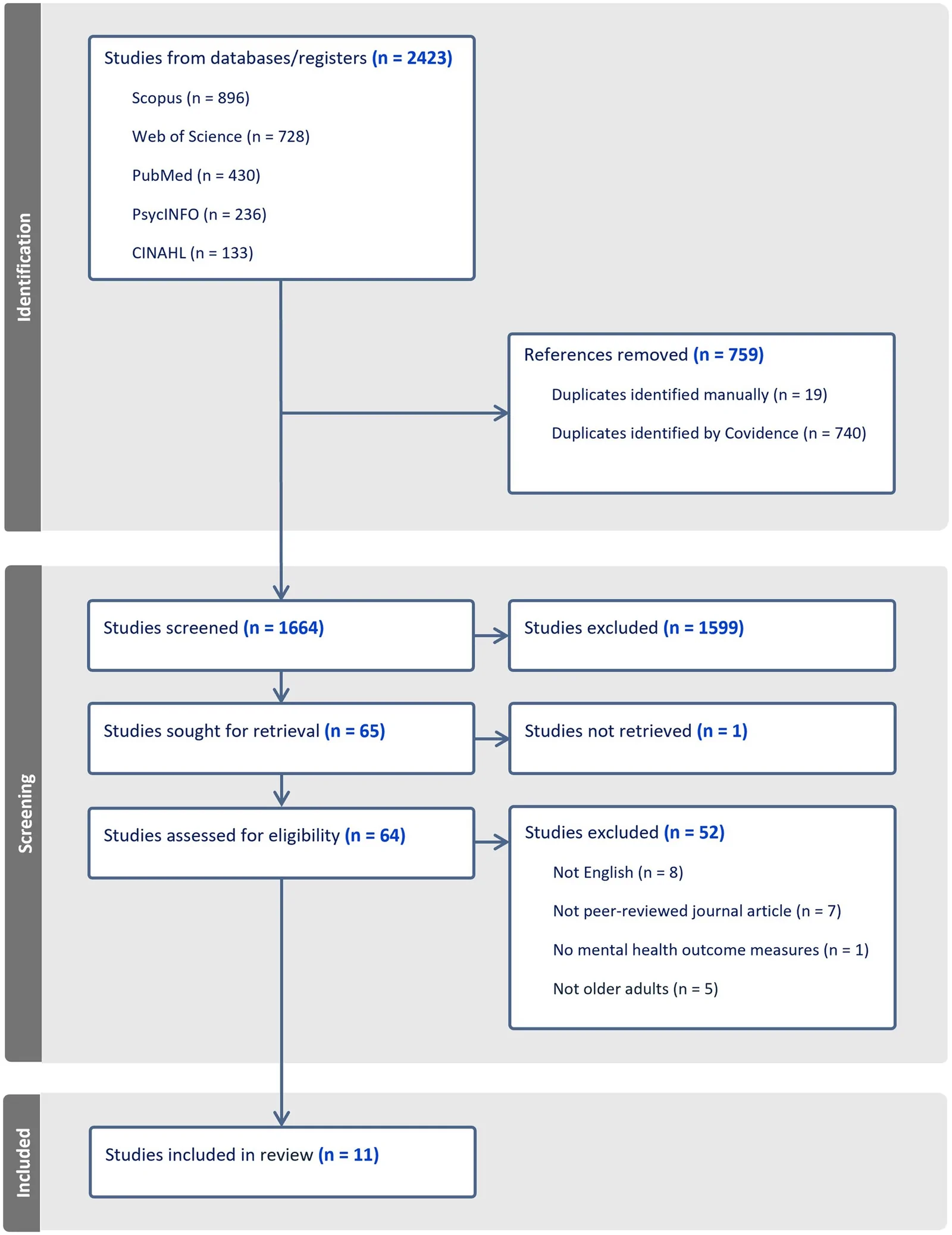
PRISMA flow diagram of the study selection process.

### Data extraction and quality assessment

Data were extracted from the included articles and coded using the program Atlas.ti. Extraction was performed by either the first author (N.K.) or a research associate (M.W.), each verifying the other’s entries and resolving disagreements by consensus discussions. Methodological quality was assessed by two independent reviewers (N.K., R.B.), using the Mixed Methods Appraisal Tool (MMAT, Hong et al., 2018). The MMAT includes five criteria for each specific study design, with appraisals of Yes, No, or Can’t tell, which were discussed until consensus was reached. No overall scores were calculated from the ratings (Hong et al., 2018). The assessments were used to describe and compare the methodological quality of the included studies, not to exclude studies.

Numerical and narrative data about intervention characteristics (i.e., target group, setting and delivery mode, dosage, activities and tailoring) were extracted and synthesized. Interventions were classified as gratitude or savoring interventions according to their primary active ingredient and guided by the terminology used by original authors. We also examined the included articles for data on acceptability (i.e., how well the intervention is received by stakeholders). Acceptability indicators included participant evaluations, adherence and continuation, potential barriers for implementation, and recommendations for future research (as described in the articles). Potential barriers and recommendations were first inductively grouped and then organized into descriptive themes by one coder (N.K.) in consensus discussions with M.W. and R.B.

With regard to the effectiveness of the interventions, we extracted the study sample information (e.g., sample size, gender, age), design, outcome measures, statistical analyses, and results. We only extracted analyses and results on the effectiveness of the intervention on mental health outcomes (i.e., well-being, psychopathology), including means and standard deviations, test statistics, significance, and effect size reported in the primary studies.

## Results

### Study characteristics

A total of 11 studies were included (Figure 1). Table 1 shows the study characteristics, intervention characteristics, and effectiveness of the included studies. The studies were published between 2011 and 2023, with most (*n *= 8) published after 2017. Studies were mostly conducted in the United States (seven), or Europe (three: Spain, England, Italy), and one study was conducted in China. The mean age of participants varied between 63 and 88 years, and all but one of the samples were predominantly female. Participants were either community dwelling (*n *= 4), residing in long-term care (*n *= 1), or their place of residence was not specified (*n *= 4). In two studies, both community and long-term care settings were included.

**Table 1:** Study characteristics, intervention characteristics, and effectiveness.

Reference	Country and target group (people with dementia)	Design and sample	Content and tailoring	Setting, delivery mode, and dosage	Adherence and continuation	Outcome measures and statistical analyses	Effectiveness
1. Bartlett and Arpin (2019)	United States Community dwelling *Independent living facilities* (Dementia n.s.)	RCT (21 measurements) Three good things (*n *= 23) No exercisecontrol (*n *= 19) *N *= 42; *F *= 80% Age *M *= 73, *SE *= 6.43 years (range n.s.)	Gratitude Three good things diary *listing three good things that happened that day and why they happened*	Individual Paper Daily, 20 days	Adherence: some not writing about good things, or fewer than three good things	Well-being Daily gratitude (GQ6) Multilevel modeling: Well-being modeled as a function of time, condition, and Time × Condition	No time effect: *t*(32) = 0.69, *p* = .498, 95% CI [−0.01, 0.01] No condition effect: *t*(34) = 0.78, *p* = .441, 95% CI [−0.53, 1.20] No interaction effect: *t*(35) = −0.53, *p* = .599, 95% CI [−0.02, 0.01]
2. Borelli et al. (2020)	United States Community dwelling and older adult living facilities (Dementia Excluded *MMSE score*)	Post-test personal savoring (*n *= 24) Relational savoring (*n *= 39) *N *= 63; *F* = 73% Age *M *= 75.74, *SD *= 8.86 years (range 60–95)	Savoring Personal savoring *reflecting on positive memory not involving other people* Relational savoring *reflecting on positive memory involving attachment figure*	Individual Face-to-face Once, 30 min	Adherence: savoring task led by professional, fidelity check of savoring words used indicated compliance*: more first-person plural pronouns for relational savoring, no difference in positive emotion words.*	Well-being positive affect (PANAS) *t*-test IV: time DV: well-being	No time effect personal savoring on positive affect *t*(22) = 0.31, *p* = .76 Time effect relational savoring on positive affect *t*(38) = 2.31, *p* = .02, T0 < T1 *(no effect size reported*)
3. Bryant et al. (2021)	United States Residence n.s., (Dementia Excluded *Human intelligence task at least 50 times approval rate >95%*)	RCT (post-test only) Savoring life lessons (*n *= 109) Neutral control (*n *= 93) *N *= 202; *F* = 60% Age *M *= 68.96, *SD *= 4.23 years (range 65–95)	Savoring Savoring life-lessons *listing and reflecting on important lessons life had taught them*	Individual Online Once, 2 min	Adherence: analysis of exercise narratives showed *more gratitude related words, but not more positive affect words in intervention group.*	Well-being Life satisfaction (SWLS) State hope (SHS1) State self-esteem (SSES) ANCOVAs IV: condition DV: well-being	No condition effects well-being: life satisfaction, *F*(1,192) = .04, *p* = .833, *η*^2^ < .001, state hope, *F*(1,192) = 2.49, *p* = .116, *η*^2^ = .006, state self-esteem, *F*(1,192) < .01, *p* = .95, *η*^2^ <.001
4. Killen and Macaskill (2015)	United Kingdom Community dwelling, *healthy, aged >60* (Dementia Excluded *Self-reported diagnosis memory impairment*)	Pre-posttest + 1 FU Paper three good things (*n *= 40) Online three good things (*n *= 48) *N *= 88; *F* = 74% Age *M *= 70.84, *SD *= 7.51 years (range 60–91)	Gratitude Three good things diary *recording three events occurring that day that seemed positive, and why they are viewed positively* Tailoring: *examples given for assistance*	Individual Self-selected Paper or online Daily, 14 days	Adherence: confirmed by diary analysis *six dimensions: self-acceptance, positive relations, autonomy, environmental mastery, purpose in life, personal growth.* But some writing about fewer than three good things Continuation: 80% did not plan to continue	Well-being Flourishing (FS) Life satisfaction (SWLS) Pos and neg affect (SPANE) Gratitude (GQ6) Psychopathology Stress (PSS10) MANOVA: IV: condition and time DV: well-being, psychopathology	No condition effect: *F*(5,69) = 2.11, *p* = .07 (*paper = online*) Time effect: *F*(10,64) = 4.04, *p* = .001, *η*^2^ = .39 for Flourishing (T0 < T1) and Stress (T0 > T1). No time effect for life satisfaction or positive and negative affect. Interaction effect: *F* (10,64) = 2.31, *p* = .02, *η*^2^ = .27 for positive and negative affect (*T1 paper > online*).
5. Lau and Cheng (2011)	China Residence n.s. *attending social centers or clubs* (Dementia n.s.)	RCT (post-test only) Three good things (*n *= 29) Hassle control (*n *= 25) Neutral control (*n *= 29) *N *= 83; *F* = 60% Age *M *= 62.7, *SD = *7.13 years (range 55–85)	Gratitude Brief good things exercise *recalling up to five gratitude-including events for which they feel grateful, thankful, or appreciative* Tailoring: *initial face-to-face explanation in groups of 10*–*15*)	Individual Face-to-face Once 15–20 min	Adherence: confirmed by analysis of listed events: *Gratitude and hassle condition 100% successful, neutral condition 30% pleasant, 13% unpleasant, 57% neutral*	Well-being Pos and neg affect (CAS) Psychopathology Death anxiety (DAS) ANOVAs: IV: condition DV: well-being, psychopathology	No condition effect on pos affect *F*(2,81) = 2.73, *p* = .07. Condition effect on neg affect *F*(2,81) = 4.83, *p* = .01, *η*^2^ = .11 *(hassle > gratitude = neutral*); death anxiety *F*(2,81) = 7.45, *p* = .001, *η*^2^ = .16. *(gratitude < (hassle = neutral*)
6. Pancini et al. (2023)	Italy Community dwelling and nursing home (Dementia Excluded *diagnosis dementia*)	Pre-posttest Savoring various (*n *= 11) *N *= 11; *F* = 55% Age *M *= 73.09, *SD *= 10.47 years (range 65–95)	Savoring Six relational savoring exercises *1. Listing three positive moments with loved one* *2. Immersing in one positive moment* *3. Listing two to three people they appreciated* *4. Writing and delivering gratitude letter* *5. Acts of kindness* *6. Recap, relaxation video* Tailored: *choice between recorded-audio or written text*	Individual Online *support available* 10 min/exercise Six exercises 3 weeks	Adherence: analysis of participants’ exercise comments indicated compliance for all except acts of kindness Evaluations: positive user experience, and useful in supporting a different perspective	Well-being Pos and neg affect (SPANE) *t*-tests IV: time DV: well-being	Time effect on pos emotions *t*(10) = −2.211, *p* = .051, T0 < T1. *(no effect size reported*) No time effect on neg affect *t*(10) = .719, *p* = .448
7. Redwine et al. (2016)	United States Residence n.s.; *diagnosed AHA/ACC classification stage B heart failure >3 months* (Dementia n.s.)	RCT (pre-mid-posttest) Three good things (*n *= 24) TAU control (*n *= 34) *N *= 58; *F* = 10% Age *M *= 66.2, *SD *= 7.58 years (range n.s.)	Gratitude Three–five good things diary *recording three to five things for which they are grateful* Tailoring: *initial face-to-face explanation*)	Individual Paper Daily, 8 weeks	Adherence: average of about 5 days journaling per week, 71% total intervention completion	Well-being Gratitude (GQ6) ANCOVA IV: condition, time DV: well-being	Interaction effect gratitude *F *= 6.0, *p* = .017, *η*^2^ = .10 (increases gratitude > TAU)
8. Salces-Cubero et al. (2019)	Spain Community dwelling *Non-institutionalized, regularly attending day centers* (Dementia Excluded *cognitive impairment*)	RCT + 1 FU Gratitude various (*n *= 36) Savoring various (*n *= 28) Optimism (*n *= 28) TAU control (*n *= 32) *N *= 124; *F* = 60% Age *M *= 69.05, *SD *= 7.78 years (range 60–89)	Gratitude and Savoring Three gratitude exercises *a. Good things: recalling and sharing three good things that happened the past 6 months;* *b. Expressing feelings and thoughts that lead to gratitude;* *c. Describing aspects of life generating well-being and gratitude* Three savoring exercises *a. Remembering, enjoying and sharing positive memory associated with personal object;* *b. 15-Min savoring walk, sharing experiences;* *c. Imagining positive events that could happen next week*	Group Face-to-face 70 min/session Four sessions	Adherence: n.s., but led by professional	Well-being Happiness (SHS2) Resilience (RS) Life satisfaction (SWLS) Pos and neg affect (PANAS) Psychopathology Depression (GADS) Anxiety (GADS) ANOVAs IV: condition, time DV: well-being, psychopathology	Interaction effect happiness *F*(6,240) = 3.882, *p* < .001, *η*^2^=.088, life satisfaction *F*(6,240) = 11.801, *p* < .001, *η*^2^ =.228: gratitude and savoring T0 < T1(=FU) *p* < .001. Interaction effect resilience *F*(6,240) = 7.887, *p* < .001, *η*^2^=.167, pos affect *F*(6,240) = 7.246, *p* < .001, *η*^2^=.153. gratitude and savoring T0 < T1, *p* < .001. Interaction effect neg affect *F*(6,240) = 2.677, *p* = .016, *η*^2^=.063: gratitude and savoring T0 > T1, *p* = .014 and *p* < .001 respectively). Interaction effect depression *F*(6,240) = 16.27, *p* < .001; *η*^2^ = .289: savoring T0 > T1, *p* = .007; optimism T0 > T1(=FU), *p* < .001). No interaction effect anxiety
9. Smith and Bryant (2019)	United States Residence n.s., (Dementia Excluded *Human intelligence task at least 50 times approval rate >95%*)	RCT (post-test only) Savoring life lessons (*n *= 95) Negative aging control (*n *= 129) No exercise control (*n *= 79) *N *= 303; *F* = 81% Age *M *= 68.12, *SD *= 6.31 years (range 60–89)	Savoring Savoring life lessons *listing and reflecting on important lessons life had taught them*	Individual Online Once, Single exercise	Adherence: confirmed by analysis of life lessons descriptions: *family and relationships; self-acceptance and independence; meaningful activities education and self-improvement; worry less; make better financial decisions; positive mindset.* *Mentions of gratitude (29%); bittersweet feelings (25%*)	Well-being Life satisfaction (one item PWI) Mood (one item) Psychopathology Anxiety (STAI) ANCOVAs IV: condition DV: well-being, psychopathology	Condition effect on life satisfaction *F*(2,291) = 4.44, *p* = .013, *η*^2^ = .030; mood *F*(2,295) = 3.03*, p* = .050, *η*^2^ = .02: savoring > (neg control = no exercise control) No condition effect on anxiety *F*(2,293) = 0.63, *p* = .531, *η*^2^ = .004.
10. Smith and Hanni (2019)	United States Community dwelling (Dementia n.s.)	Pre-posttest + 2 FU Savoring (*n *= 111) *N *= 111; *F* = 83% Age *M *= 70.7, *SD *= 7.2 years (range 60–93)	Savoring Savoring positive experiences *Thinking of something positive in the past, present or future, noticing the positive feelings, and appreciating the experience*	Individual Paper or online Daily, 5 min morning; 5 min evening 1 week	Adherence: 46% completed full intervention, self-reported average of 5.5 days per week doing exercise. Continuation: 1 month 82% continued activity; 3 months 59% continued activity	Well-being Happiness (SHS2) Resilience (BRS) Psychopathology Depression (CES-D) Multilevel modeling: Well-being and psychopathology modeled as a function of time, intervention fidelity (0–5 days vs 6–7 days), and time × fidelity	No interaction effect happiness Estimate = .07, *SE* = .04, *p* = .085, but increase for high fidelity *b* = .11, *SE* = .05, *p* = .018, not for low fidelity *b *= −.02, *SE* = .06, *p* = .726 Interaction effect resilience: Estimate = .08, *SE* = .03, *p* = .01: increase for high fidelity *b* = .14, *SE* = .04, *p* < .001, not for low fidelity *b *= −.01, *SE* = .05, *p* = .837 Interaction effect depression: Estimate = −.03, *SE* = .01, *p* = .041: decrease for high fidelity *b *= −.04*, SE* = .02, *p* = .025, not for low fidelity *b* = .02, *SE* = .02*, p* = .392
11. Sunding (2015)	United States Permanent residents of long-term care facility (Dementia Included *diagnosis* *dementia, cognitive impairment*)	Pre-mid-posttest (21 measurements) One good thing (*n *= 29) *N = *29; *F* = 79% Age *M *= 87.7 years, *SD =* n.s. (range 65–99)	Gratitude Gratitude dinner intervention *sharing something they are thankful for or a good thing that happened that day*	Group Face-to-face Daily 10 min/day 7 days	Adherence: checked by experimenter through observation on day 1, and an unannounced day	Well-being Self-developed scale (nine items) ANOVAs IV: time DV: well-being	Time effect well-being *F*(2,56) = 9.09, *p* < .001. ((T0 = T1) < T3)

*Note*. References in the table are the papers included in the review; see reference list. ANCOVA = analysis of covariance; ANOVA = analysis of variance; *b* = regression coefficient; BRS = Brief Resilience Scale; CAS = Chinese Affect Scale; CES-D = Center for Epidemiologic Studies Depression Scale; DAS = Death Anxiety Scale; DV = dependent variable; FS = Flourishing Scale; FU = follow-up; GADS = Goldberg Anxiety and Depression Scale; GQ-6 = Gratitude Questionnaire; IV = independent variable; MANOVA = multivariate analysis of variance; MMSE = Mini-Mental State Examination; n.s. = not specified; PANAS = Positive and Negative Affect Schedule; PSS-10 = Perceived Stress Scale; PWI = Personal Wellbeing Index; RS = Resilience Scale; SHS1 = State Hope Scale; SHS2 = Subjective Happiness Scale; SPANE = Scale of Positive and Negative Experience; SSES = State Self-Esteem Scale; STAI = State-Trait Anxiety Inventory; SWLS = Satisfaction With Life Scale; TAU = treatment as usual; T0–T3 = measurement time points (e.g., baseline, follow-up); *η*² = effect size.

Only one study specifically included people living with dementia (Sunding, 2015). Therefore, we were unable to explore acceptability and effectiveness for people with and without dementia. Others excluded people with dementia, memory problems, or cognitive impairment (*n *= 6), or did not state anything about this in the inclusion criteria (*n *= 4). Reasons for exclusion were reported as “*the intervention required daily reflections*” (Killen & Macaskill, 2015, p. 952), and “*due to concerns about their ability to identify and expand upon memories*” (Borelli et al., 2020, p. 335).

#### Quality assessment

The quality assessments of included studies using the MMAT (Table S2) showed variation in terms of methodological compliance and reporting. None of the studies had “perfect” quality, as reporting was often insufficient. The included randomized controlled trial (RCT) studies (*n *= 6) appropriately performed randomization, but often failed to report the randomization method. When comparability of groups at baseline was reported (*n *= 2 studies), both comparable and non-comparable groups were described. The blinding of outcome assessors was impossible with self-reported mental health measures. The pre-post studies (*n *= 5 studies) used appropriate measures, except for one non-validated well-being scale. Two studies accounted for confounders, and only one reported representativeness of participants (describing aspects of how their sample was not representative). Outcome data varied from complete outcome data (*n *= 3 RCT studies; *n *= 3 pre-post studies), to high exclusion or drop-out rates (*n *= 2 RCT studies), or exact drop-out rates were unclear (*n *= 3 studies). Finally, studies reported that participants adhered to the intervention (*n *= 3 RCT studies), or the intervention was administered as intended (*n *= 3 pre-post studies), or this was not clearly reported (*n *= 5 studies).

### Review question 1: characteristics of gratitude and savoring interventions for older adults

Five studies examined a gratitude intervention (Bartlett & Arpin, 2019; Killen & Macaskill, 2015; Lau & Cheng, 2011; Redwine et al., 2016; Sunding, 2015), five studies examined savoring positive things (Borelli et al., 2020; Bryant et al., 2021; Pancini et al., 2023; Smith & Bryant, 2019; Smith & Hanni, 2019), and one study compared gratitude and savoring interventions (Salces-Cubero et al., 2019) (Table 1). Individual interventions (*n *= 9) were delivered online (*n *= 3), on paper (*n *= 2), a combination (*n *= 2), or face-to-face (*n *= 2). Group interventions (*n *= 2) were face-to-face. Dosage varied between performing a single exercise once for 2–30 min (*n *= 4), to repeating one exercise daily for 1–8 weeks (*n *= 5), or performing various exercises during six 10-min sessions (*n *= 1), or during four 70-min sessions (*n *= 1). The content of the interventions was not specifically designed for the older adult population. Several interventions did offer some form of assistance: providing an initial face-to-face explanation of the exercise (Redwine et al., 2016), offering both recorded-audio or written text (Pancini et al., 2023), or providing specific examples for inspiration (Killen & Macaskill, 2015).

#### Gratitude interventions

Gratitude journaling was included most often (*n *= 4), by recording three to five good things that happened that day and reasoning why those things happened, based on the instructions of Emmons and McCullough (2003). A variation of this exercise was used in long-term care, with staff members starting daily group dinners by asking residents to share something they were thankful for or something good that happened that day (Sunding, 2015). This included people living with dementia. One multi-exercise gratitude group intervention included sharing the good things in life that had occurred in the last 6 months, expressing the feelings and thoughts that led to gratitude, and describing current aspects of life that generated gratitude (based on Lomas et al., 2014) (Salces-Cubero et al., 2019).

#### Savoring interventions

Savoring interventions had diverse subject matter (savoring in general, relationships, and life lessons). Savoring in general (*n *= 2) could be induced by focusing on the present, the past, and the future: “*something good that’s happening right now, a pleasant memory, or something positive that will happen in the future*” (Smith & Bryant, 2019). Relational savoring (*n *= 2) was induced by discussing sensory details, emotions, thoughts, and meanings associated with memories with attachment figures (Borelli et al., 2020), or with a combination of six exercises focused on the past, present, and future (Pancini et al., 2023). It should be noted that the latter was presented as relational savoring, but also seemed to include two gratitude exercises (i.e., listing two to three people they were grateful for, and a gratitude visit). Savoring life lessons (*n *= 2) included looking back and reflecting on lessons learned in life, selecting the most valuable lesson, and imagining life without that lesson. Finally, one multi-exercise gratitude group intervention included savoring memories connected to a personal object, a 15-min savoring walk, and imagining positive events next week (based on Smith et al., 2014; Salces-Cubero et al., 2019).

### Review question 2: acceptability of gratitude and savoring interventions for older adults

#### Participant evaluations

Only two studies included participant intervention evaluations: exploring participant preferences of online versus paper delivery (Killen & Macaskill, 2015), and user experience and perceived usefulness of an online intervention (Pancini et al., 2023). The latter indicated positive user experience (*n *= 11 participants), and users felt the intervention was effective for adopting a different perspective and focus on positive aspects in life (Pancini et al., 2023).

#### Adherence and continuation

Adherence was addressed in various ways in 10 of the studies based on analyses of exercise and diary descriptions (*n *= 8), self-reports (*n *= 1), or experimenter check (*n *= 1, Table 1). Several studies indicated they (partly) confirmed adherence (*n *= 6), with full completion ranging from 46% of participants (Smith & Hanni, 2019) to 71% of participants (Redwine et al., 2016). Suboptimal adherence included not writing about good things, or fewer than three good things (Bartlett & Arpin, 2019; Killen & Macaskill, 2015). Continuation of exercises was addressed in two studies. Most participants in the first study reported continued savoring of positive experiences (82% at 1 month, 59% at 3 months; Smith & Hanni, 2019), while in the second a majority did not intend to continue gratitude journaling (80%, Killen & Macaskill, 2015).

#### Potential barriers and recommendations for implementation and research

All studies addressed potential barriers and recommendations for implementation and effectiveness of the interventions. Four main themes regarding potential barriers and recommendations were identified: target group, setting and delivery mode, dosage, and content and tailoring.

##### Target group

The studies described difficulty recruiting community-dwelling older adults (Bartlett & Arpin, 2019) and recommended identifying characteristics of older adults who would benefit most (Redwine et al., 2016; Smith & Hanni, 2019). The need to take a more diverse sample into account was stressed in eight of the included studies, including more males, more advanced age groups, people with lower education levels and digital competence, and with various cultural ethnic/racial backgrounds and spiritual beliefs (Borelli et al., 2020; Bryant et al., 2021; Lau & Cheng, 2011; Pancini et al., 2023; Smith & Bryant, 2019: Smith & Hanni, 2019; Sunding, 2015). Potential suitability for use in clinical samples was acknowledged (Lau & Cheng, 2011).

##### Setting and delivery mode

Recommendations regarding setting and delivery mode included using online delivery while accommodating declined vision, hearing, and psychomotor functioning (Killen & Macaskill, 2015), as well as group-based delivery to capitalize on social interactions (Smith & Bryant, 2019). The potential of gratitude and savoring exercises to complement broader interventions was highlighted (e.g., for interventions aimed at improving physical exercise compliance, or perceptions of aging; Redwine et al., 2016; Smith & Bryant, 2019). Additional recommendations concerned using alternative comparison groups: including an active or neutral control task, or comparing independently administered activities (Bryant et al., 2021; Salces-Cubero et al., 2019; Sunding, 2015).

##### Dosage

Six studies indicated that short interventions could have limited effects, or recommended to prolong the duration, with repeated and long-term exposures so that the practice of gratitude and savoring could become a way of life (Bryant et al., 2021; Killen & Macaskill, 2015; Lau & Cheng, 2011; Redwine et al., 2016; Salces-Cubero et al., 2019; Sunding, 2015). Almost all studies stressed the need for assessing longer-term benefits of the interventions in a large-scale controlled study (Bartlett & Arpin, 2019; Borelli et al., 2020; Bryant et al., 2021; Killen & Macaskill, 2015; Lau & Cheng, 2011; Pancini et al., 2023; Redwine et al., 2016; Smith & Bryant, 2019; Smith & Hanni, 2019; Sunding, 2015).

##### Content and tailoring

It was recommended to further investigate the need for age-specific amendments to intervention exercise instructions. For gratitude exercises, potential barriers included boredom, and the inability to identify three examples per day (Bartlett & Arpin, 2019; Killen & Macaskill, 2015), and negative feelings, such as guilt or indebtedness when reflecting on gratitude (Killen & Macaskill, 2015; Pancini et al., 2023). It was both recommended to increase flexibility (in the number and frequency of entries, Killen & Macaskill, 2015), and to decrease flexibility (fill in the blanks, Bartlett & Arpin, 2019). For savoring exercises, it was suggested that older adults may no longer be motivated to savor the same positive experiences in the same way as they were younger (Pancini et al., 2023). Studies recommended examining the most effective savoring focus (past, present, or future) for this population, as well as the effects of different types of savoring (e.g., attachment-based, achievement-based, nondirective savoring) (Borelli et al., 2020; Salces-Cubero et al., 2019; Smith & Hanni, 2019).

### Review question 3: effectiveness of gratitude and savoring interventions for older adults

The included studies had 1,114 participants combined (range 11–303), with most participants in the control groups (*n *= 468) or receiving savoring interventions (*n *= 417 participants), compared to gratitude interventions (*n *= 229, Table 1). Two studies excluded data of noncompliant participants in their effectiveness analyses (Bryant et al., 2021; Smith & Bryant, 2019). Effect sizes and metrics of the primary studies can be found in Table 1.

Gratitude and savoring interventions showed similar effects on mental health. Of the six studies that included gratitude exercises, most reported medium to large effects on well-being (*n *= 4: Killen & Macaskill, 2015; Redwine et al., 2016; Salces-Cubero et al., 2019; Sunding, 2015), and psychopathology (*n *= 3: Killen & Macaskill, 2015; Lau & Cheng, 2011; Salces-Cubero et al., 2019). Of the six studies that examined savoring, most reported small to large effects on well-being (*n *= 5: Borelli et al., 2020; Pancini et al., 2023; Salces-Cubero et al., 2019; Smith & Bryant, 2019; Smith & Hanni, 2019), and medium to large effects on psychopathology (*n *= 2: Salces-Cubero et al., 2019; Smith & Hanni, 2019). One study including both savoring and gratitude interventions reported improvements across most outcome measures for the savoring condition, but this was based on within-group changes, not on between-group comparisons (Salces-Cubero et al., 2019).

Six studies used a randomized controlled trial (RCT) design, and five had a single-arm, pre-post design. Similar effectiveness was observed across both types of studies: most reported effects on all outcomes (*n *= 2 RCTs, *n *= 2 pre-post studies), or a subset of the outcomes (*n *= 2 RCTs, *n *= 3 pre-post studies), although two RCT studies found no effects (i.e., Bartlett & Arpin, 2019; Bryant et al., 2021).

Outcome measures of mental health varied widely across studies. Well-being was measured in all 11 studies (e.g., positive and negative affect, life satisfaction, happiness, and gratitude, see Table 1), compared to five studies including measures of psychopathology (e.g., depression and anxiety, stress, and loneliness). Similar effects were reported for both types of outcome measures, with most studies reporting effects on all included outcome measures (*n *= 6 for well-being, *n *= 3 for psychopathology), or a subset of included outcome measures (*n *= 2 for well-being, *n *= 1 for psychopathology). For well-being, some studies found no effect on gratitude, positive affect, life satisfaction, hope or self-esteem (Bartlett & Arpin, 2019; Bryant et al., 2021; Lau & Cheng, 2011). For psychopathology, two studies found no effect on anxiety (Salces-Cubero et al., 2019; Smith & Bryant, 2019).

When comparing one-time exercises (four studies), daily exercises (five studies), and multi-session formats (two studies), no consistent patterns emerged: findings were mixed across all intervention types, including no effects, effects on only certain outcomes, and effects across multiple outcomes, with effect sizes ranging from small to large. However, one pre-post study showed that the three-good things exercise was similarly effective online compared to paper based (Killen & Macaskill, 2015), and two pre-post studies showed only an effect of certain kinds of savoring (relational savoring, not general savoring; Borelli et al., 2020), or was only effective for people who completed the exercise for 6 or 7 days (compared to <6 days; Smith & Hanni, 2019).

## Discussion and implications

This narrative review highlights the emerging field of gratitude and savoring research among older adults, presenting preliminary evidence that while (1) intervention characteristics vary considerably, they (2) could potentially be acceptable for older adults with relatively preserved cognitive function, and (3) they may be effective for improving mental health in this population of older adults. Overall, gratitude and savoring interventions showed broadly similar effects on mental health, although the current evidence is insufficient to directly compare these approaches. Across studies, findings were heterogeneous and there was a wide variation in outcome measures, and no consistent patterns were found across study designs, type of outcome measure (well-being or psychopathology) or intervention formats. Cautious interpretation of these results is required due to the limited number of included studies, the specific population of predominantly community-dwelling older adults without significant cognitive impairment, along with inconsistencies in reporting quality and methodological limitations in several primary studies (e.g., small sample sizes, high attrition rates, use of a non-validated measure).

Despite these constraints, the gratitude and savoring interventions in the present study provide preliminary evidence of their potential effectiveness in improving well-being and reducing psychopathology among older adults with higher levels of cognitive functioning. This is in line with prior meta-analytic evidence showing that gratitude and savoring interventions can lead to improvements in well-being and reductions in psychopathology across various populations, although the magnitude and consistency of these benefits show some variability (Cregg & Cheavens, 2021; Cullen et al., 2024; Davis et al., 2016; Dickens, 2017; Diniz et al., 2023; Renshaw & Steeves, 2016; Zheng et al., 2025). This can be understood through the broaden-and-build theory of positive emotions (Fredrickson, 2013). Gratitude and savoring exercises elicit positive emotions that broaden attention and cognitive flexibility, and over time help build enduring psychological and social resources, contributing to improved mental health. It is surprising that half of the studies did not assess positive affect, and two found no effects (Killen & Macaskill, 2015; Lau & Cheng, 2011), although others did report improvements. None, however, tested the full broaden-and-build mechanism. In later life, these accumulated resources may be especially valuable in buffering age-related stressors such as loss, health decline, or reduced social networks. Importantly, in the absence of comprehensive process evaluations, it remains unclear to what extent gratitude and savoring interventions should be tailored to older adults regarding content, optimal dosage, and delivery mode, and whether they require further adaptation to meet the needs of specific subgroups of older adults, such as individuals with cognitive decline.

Concerning intervention content, the included studies did not tailor their interventions to the older adult population, with gratitude exercises primarily involving journaling or listing, and savoring exercises comprising of varied timeframes (i.e., past, present, future) and topics (e.g., general, relationships, life lessons). It was suggested by the authors of included studies that older adults may benefit more from focusing on the past and present rather than the future (Borelli et al., 2020; Salces-Cubero et al., 2019) which aligns with the growing body of research that has explored positive reminiscence and positive life review (Pinquart, 2024; Zhong et al., 2023). However, appreciating and savoring the present and (near) future may offer additional benefits, such as fostering a sustained sense of hope, purpose, and life direction (Bryant et al., 2021; Webster, 2016), which remain relevant for older adults (Ferguson et al., 2017; Pfund et al., 2026).

There was some indication that the thematic focus of the exercises included in this narrative review may have been differentially effective in older adults. For instance, reflecting on one’s morning routine (intended as a neutral control) induced savoring (Bryant et al., 2021), and only relational savoring, not personal savoring, was effective in one study (Borelli et al., 2020). These findings can be interpreted through socioemotional selectivity theory, which posits that older adults increasingly prioritize emotionally meaningful experiences (Carstensen et al., 1999). As a result, even everyday activities such as a morning routine may carry positive emotional significance and elicit savoring, while relational content may be particularly effective due to the increased importance of close relationships. Similar patterns have been observed with gratitude exercises, where interpersonal gratitude exercises (gratitude texts and letters) were more effective than intrapersonal exercises (gratitude lists, mental subtraction tasks; Dang et al., 2026), yet less often examined (Cregg & Cheavens, 2021).

Regarding optimal dosage, the intervention dosage in the included studies varied considerably, ranging from doing a single exercise once for 2 min, to doing four 70-min sessions. Increasing the intensity of the interventions was recommended to promote the practice of gratitude and savoring as a way of life. There is some indication that long-form gratitude writing exercises are more effective than lists to improve well-being (Regan et al., 2023). On the other hand, intervention length did not mediate the results of expressed gratitude interventions in a recent meta-analysis of RCTs (Kirca et al., 2023). It is important to take into account the potential impact of boredom and the challenge of continuously generating new, positive, and personally meaningful examples in this population.

With respect to delivery mode, most of the gratitude and savoring interventions were delivered individually, and either on paper or online, and no consistent patterns emerged across intervention formats. Although group-based delivery was often recommended by the authors of primary studies, no studies assessed whether group-based delivery was preferred by the older users or had a superior effect. Furthermore, only a few included studies used a face-to-face format, which may improve effectiveness (Carr et al., 2021), but at the cost of reduced flexibility and scalability. This highlights a broader challenge in determining optimal delivery modes, particularly given the limited evidence on implementing and scaling eHealth interventions for older adults (Buyl et al., 2020). The current review suggests that online delivery is potentially acceptable and feasible for older adults (Pancini et al., 2023), with results comparable to paper-based delivery (Killen & Macaskill, 2015). However, this may vary across subgroups of older adults, as online delivery must account for barriers such as digital literacy (which was relatively high in the included studies), as well as fears, limited self-efficacy and motivation, and physical and cognitive limitations (Wilson et al., 2021).

Notably, specific subgroups of older adults remain underrepresented in this line of research, including the oldest-old, men, individuals with lower socioeconomic status, and racially and ethnically diverse populations. This also applies to people living with dementia, who constitute a significant and increasing proportion of older adults (WHO, 2017), but who were only considered in one of the included studies (Sunding, 2015). Previous research has indicated that multi-component PPI studies including gratitude and savoring exercises were feasible and effective for people with mild cognitive impairment, which can be a precursor for dementia (Tsiflikioti et al., 2023; Yao et al., 2023). Concerns about diminished learning abilities and the capacity to identify and elaborate on memories were provided as reasons for excluding people living with cognitive decline (e.g., Borelli et al., 2020), yet this has inevitably led to underrepresentation of this population in PPI research. People with dementia have positive life experiences (Kloos et al., 2024; Miklitz et al., 2024; Wolverson et al., 2016), which can be appreciated and savored.

Taken together, future research should explore the feasibility, acceptability, and effectiveness of gratitude and savoring interventions across more diverse subgroups, including older adults at various stages of cognitive decline. To better understand how these interventions work in older adults, there is a need to examine how the temporal and thematic focus of these exercises influence their effectiveness and suitability for this population, for example through designs that systematically vary timeframe (past, present, future) and content within a single study (Bryant et al., 2021). Given that only two included studies incorporated formal participant evaluations, future research should also prioritize user-centered approaches to better capture intervention acceptability. Incorporating the experiences and preferences of older adults in the development and evaluation of interventions may help ensure person–exercise fit (Lyubomirsky & Layous, 2013) and better meet individual needs (Niclasen et al., 2019). Approaches such as co-design and structural integration of participant evaluations could, in turn, provide valuable insights into the optimal content, dosage, and delivery modes for this heterogeneous population.

### Strengths and limitations

This narrative review contributes to the PPI literature for older adults, who have been historically overlooked in this field. To the best of our knowledge, this is the first review to specifically examine both gratitude and savoring interventions in older adults, including populations with and without cognitive impairment, across a range of study designs. The conceptual overlap between gratitude and savoring was reflected in the similarity in exercises (e.g., gratitude practices in a relational savoring intervention, Pancini et al., 2023), similar effects on well-being, but also in the similar qualitative themes found in exercise descriptions of the included studies. This underscores the importance and rationale for considering gratitude and savoring together. Future research should, however, further explore the conceptual and empirical commonalities to determine whether they should be considered distinct constructs or integrated within a unified framework.

A strength of this study is that, in addition to intervention characteristics and effectiveness, acceptability was also considered, highlighting the current lack of comprehensive process evaluations. Acceptability was assessed using a combination of direct participant evaluations, adherence and continuation rates, and reported barriers and recommendations. However, as indirect measures such as adherence cannot fully capture users’ subjective experiences, conclusions regarding acceptability remain tentative.

The limited number and heterogeneity of included studies further limits the conclusions that can be drawn, but simultaneously highlights that older adults are an underserved population in PPI research. Only peer-reviewed articles written in English that examined solely gratitude or savoring for older adults were eligible for this review. This led to the exclusion of eight articles written in other languages (four were Spanish), as well as 25 multi-component PPIs. While combining various PPIs into one multi-component intervention may improve their effectiveness (Carr et al., 2021), this complicates the ability to isolate the feasibility, acceptability, and effectiveness of specific gratitude and savoring exercises. Finally, generalizability is limited as the included studies were mainly conducted in western countries among relatively young older adults who were predominantly female, well-educated, community-dwelling and without cognitive impairment.

## Conclusion

The gratitude and savoring exercises examined in this narrative review could potentially be acceptable for older adults with higher levels of cognitive functioning, and may be effective for improving mental health in this population, although current evidence does not allow for robust conclusions. The need for tailoring these interventions to older adults should be investigated further in terms of dose, delivery method, and content. Importantly, the potential applicability for more diverse groups of older adults, including the growing number of people living with dementia, should be explored.

## Supplementary Material

gnag206_Supplementary_Data

## Data Availability

The data that support this study are available upon reasonable request. The data included in this systematic review are secondary and all original data are available in the published articles listed in the reference list. This study was pre-registered in PROSPERO (registration record CRD42023487496).
