# Supplementary material for: Gratitude and savoring interventions for older adults: a narrative review
Source: Gerontologist. 2026 Aug 27;66(11):gnag206. doi: 10.1093/geront/gnag206 (PMC13658281; doi:10.1093/geront/gnag206)
Supplement: Supplementary file 1 [file gnag206_supplementary_data.docx]

**Title: Gratitude and savoring interventions for older adults: a narrative review**

**Supplementary Table 1**

*Search string per database*

| **Database** | **#hits** | **Search string** |
| --- | --- | --- |
| Cinahl | 121 | S1. TI gratitude OR grateful* OR thankful* OR "count* W2 bless*" OR appreciat* OR "Positive Psycholog*" OR "three good things" OR savor* OR savour* 4401  S2. TI ( intervention OR training OR treatment OR program OR therap* OR psychotherapy* OR exercise* OR promot* OR practice* OR diary OR diaries OR journal* OR letter* OR list* OR writing* ) OR AB ( intervention OR training OR treatment OR program OR therap* OR psychotherapy* OR exercise* OR promot* OR practice* OR diary OR diaries OR journal* OR letter* OR list* OR writing* ) 2881419  S3. TI ( aged OR aging OR ageing OR "advanced age" OR elder* OR geriatric* OR gerontology* OR "old* adult*" OR "old* patient*" OR "old* people" OR "old* person*" OR "oldest old" OR "very old" OR "extreme old" OR "old* subject*" OR "old age*" OR senior* OR dement* OR alzheimer* OR "Cognitive impairment" OR "memory loss" ) OR AB ( aged OR aging OR ageing OR "advanced age" OR elder* OR geriatric* OR gerontology* OR "old* adult*" OR "old* patient*" OR "old* people" OR "old* person*" OR "oldest old" OR "very old" OR "extreme old" OR "old* subject*" OR "old age*" OR senior* OR dement* OR alzheimer* OR "Cognitive impairment" OR "memory loss" ) 573647  S4. S1 AND S2 AND S3 121 |
|  |  |  |
| Psycinfo | 207 | 1 (gratitude or grateful* or thankful* or "count* W2 bless*" or appreciat* or "Positive Psycholog*" or "three good things" or savor* or savour*).ti. 6143  2 (aged or aging or ageing or "advanced age" or elder* or geriatric* or gerontology* or "old* adult*" or "old* patient*" or "old* people" or "old* person*" or "oldest old" or "very old" or "extreme old" or "old* subject*" or "old age*" or senior* or dement* or alzheimer* or "Cognitive impairment" or "memory loss").ti. or (aged or aging or ageing or "advanced age" or elder* or geriatric* or gerontology* or "old* adult*" or "old* patient*" or "old* people" or "old* person*" or "oldest old" or "very old" or "extreme old" or "old* subject*" or "old age*" or senior* or dement* or alzheimer* or "Cognitive impairment" or "memory loss").ab. 569564  3 (intervention or training or treatment or program or therap* or psychotherapy* or exercise* or promot* or practice* or diary or diaries or journal* or letter* or list* or writing*).ti. or (intervention or training or treatment or program or therap* or psychotherapy* or exercise* or promot* or practice* or diary or diaries or journal* or letter* or list* or writing*).ab. 2160998  4 1 and 2 and 3 207 |
| Pubmed | 356 | ("gratitude"[Title] OR "grateful*"[Title] OR "thankful*"[Title] OR "count bless"[Title:~2] OR "appreciat*"[Title] OR "positive psycholog*"[Title/Abstract] OR "three good things"[Title] OR "savor*"[Title] OR "savour*"[Title])  AND ("intervention"[Title/Abstract] OR "training"[Title/Abstract] OR "treatment"[Title/Abstract] OR "program"[Title/Abstract] OR "therap*"[Title/Abstract] OR "psychotherapy*"[Title/Abstract] OR "exercise*"[Title/Abstract] OR "promot*"[Title/Abstract] OR "practice*"[Title/Abstract] OR "diary"[Title/Abstract] OR "diaries"[Title/Abstract] OR "journal*"[Title/Abstract] OR "letter*"[Title/Abstract] OR "list*"[Title/Abstract] OR "writing*"[Title/Abstract])  AND ("aged"[Title/Abstract] OR "aging"[Title/Abstract] OR "ageing"[Title/Abstract] OR "advanced age"[Title/Abstract] OR "elder*"[Title/Abstract] OR "geriatric*"[Title/Abstract] OR "gerontology*"[Title/Abstract] OR "old adult"[Title/Abstract] OR “old adults”[Title/Abstract] OR “older adult”[Title/Abstract] OR “older adults”[Title/Abstract] OR “oldest adult”[Title/Abstract] OR “oldest adults”[Title/Abstract] OR "old patient"[Title/Abstract] OR "old people"[Title/Abstract] OR "old person"[Title/Abstract] OR "oldest old"[Title/Abstract] OR "very old"[Title/Abstract] OR "extreme old"[Title/Abstract] OR "old subject"[Title/Abstract] OR "old age"[Title/Abstract] OR “old aged”[Title/Abstract] OR “older age”[Title/Abstract] OR “older aged”[Title/Abstract] OR “older ages”[Title/Abstract] OR “older aging”[Title/Abstract] OR “older ageing”[Title/Abstract] OR “oldest age”[Title/Abstract] OR “oldest ages”[Title/Abstract] OR “oldest aged”[Title/Abstract] OR “oldest aging”[Title/Abstract] OR “oldest ageing”[Title/Abstract] OR “senior*”[Title/Abstract] OR "dement*"[Title/Abstract] OR "alzheimer*"[Title/Abstract] OR "Cognitive impairment"[Title/Abstract] OR "memory loss"[Title/Abstract]) |
| Scopus | 780 | ( TITLE ( gratitude OR grateful* OR thankful* OR appreciat* OR "Positive Psycholog*" OR (count* W/2 bless*) OR "three good things" OR savor* OR savour* )  AND TITLE-ABS-KEY ( intervention OR training OR treatment OR program OR therap* OR psychotherapy* OR exercise* OR promot* OR practice* OR diary OR diaries OR journal* OR letter* OR list* OR writing* )  AND TITLE-ABS-KEY ( aged OR aging OR ageing OR "advanced age" OR elder* OR geriatric* OR gerontology* OR "old* adult*" OR "old* patient*" OR "old* people" OR "old* person*" OR "oldest old" OR "very old" OR "extreme old" OR "old* subject*" OR "old age*" OR “senior*” OR dement* OR alzheimer* OR "Cognitive impairment" OR "memory loss" ) ) |
| Web of Science | 601 | 1. TI=(gratitude OR grateful* OR thankful* OR "count* NEAR/2 bless*" OR appreciat* OR "Positive Psycholog*" OR "three good things" OR savor* OR savour* ) 16871  2. TS=(aged OR aging OR ageing OR "advanced age" OR elder* OR geriatric* OR gerontology* OR "old* adult*" OR "old* patient*" OR "old* people" OR "old* person*" OR "oldest old" OR "very old" OR "extreme old" OR "old* subject*" OR "old age*" OR senior* OR dement* OR alzheimer* OR "Cognitive impairment" OR "memory loss") 5024282  3. TS=(intervention OR training OR treatment OR program OR therap* OR psychotherapy* OR exercise* OR promot* OR practice* OR diary OR diaries OR journal* OR letter* OR list* OR writing*) 16923048  4. #1 AND #2 AND #3 601 |

**Supplementary Table 2.** *Quality assessment of included RCT’s and pre-post studies using the MMAT*

| **RCT studies** | | | **2.1 Is randomization appropriately performed?** | | **2.2 Are groups comparable at baseline?** | **2.3 Are there complete outcome data?** | **2.4 Are outcome assessors blinded to the intervention provided?** | | **2.5 Did the participants adhere to the assigned intervention?** | |  |
| --- | --- | --- | --- | --- | --- | --- | --- | --- | --- | --- | --- |
| 1 | Bartlett & Arpin (2019) | Can’t tell | | Can’t tell | | Yes | | No | | Can’t tell | |
| 3 | Bryant et al. (2021) | Yes | | Can’t tell | | No | | No | | Yes | |
| 5 | Lau & Cheng (2011) | Can’t tell | | Can’t tell | | Yes | | No | | Yes | |
| 7 | Redwine et al. (2017) | Yes | | Yes | | No | | Can’t tell | | Can’t tell | |
| 8 | Salces-Cubero et al. (2018) | Can’t tell | | Can’t tell | | Yes | | No | | Can’t tell | |
| 10 | Smith & Hanni (2019) | Yes | | No | | Can’t tell | | No | | Yes | |
| **Pre-posttest studies** | | | **3.1 Are the participants representative of the target population?** | | **3.2 Are measurements appropriate regarding both the outcome and intervention (or exposure)?** | **3.3. Are there complete outcome data?** | **3.4 Are the confounders accounted for in the design and analysis?** | | **3.5. During the study period, is the intervention administered (or exposure occurred) as intended?** | |  |
| 2 | Borelli et al. (2020) | Can’t tell | | Yes | | Yes | | Yes | | Yes | |
| 4 | Killen & Macaskill (2015) | No | | Yes | | Yes | | Can’t tell | | Yes | |
| 6 | Pancini et al. (2023) | Can’t tell | | Yes | | Can’t tell | | Can’t tell | | Yes | |
| 9 | Smith & Bryant (2019) | Can’t tell | | Yes | | Yes | | Yes | | Can’t tell | |
| 11 | Sunding (2015) | Can’t tell | | No | | Can’t tell | | Can’t tell | | Can’t tell | |
